# Attentional capture is modulated by stimulus saliency in visual search as evidenced by event-related potentials and alpha oscillations

**DOI:** 10.3758/s13414-022-02629-6

**Published:** 2022-12-16

**Authors:** Norman Forschack, Christopher Gundlach, Steven Hillyard, Matthias M. Müller

**Affiliations:** 1 Experimental Psychology and Methods, Wilhelm Wundt Institute for Psychology, University of Leipzig, Leipzig, Germany; 2 University of California, San Diego, and Leibniz Institute of Neurobiology, Magdeburg, Germany

**Keywords:** Electrophysiology, Cognitive and attentional control, Attentional capture

## Abstract

This study used a typical four-item search display to investigate top-down control over attentional capture in an additional singleton paradigm. By manipulating target and distractor color and shape, stimulus saliency relative to the remaining items was systematically varied. One group of participants discriminated the side of a dot within a salient orange target (ST group) presented with green circles (fillers) and a green diamond distractor. A second group discriminated the side of the dot within a green diamond target presented with green circle fillers and a salient orange square distractor (SD group). Results showed faster reaction times and a shorter latency of the N2pc component in the event-related potential (ERP) to the more salient targets in the ST group. Both salient and less salient distractors elicited Pd components of equal amplitude. Behaviorally, no task interference was observed with the less salient distractor, indicating the prevention of attentional capture. However, reaction times were slower in the presence of the salient distractor, which conflicts with the hypothesis that the Pd reflects proactive distractor suppression. Contrary to recent proposals that elicitation of the Pd requires competitive interactions with a target, we found a greater Pd amplitude when the distractor was presented alone. Alpha-band amplitudes decreased during target processing (event-related desynchronization), but no significant amplitude enhancement was observed at electrodes contralateral to distractors regardless of their saliency. The results demonstrate independent neural mechanisms for target and distractor processing and support the view that top-down guidance of attention can be offset (counteracted) by relative stimulus saliency.

**Supplementary Information:**

The online version contains supplementary material available at 10.3758/s13414-022-02629-6.

## Introduction

From everyday experience, we know that salient stimuli like a flashing warning sign or a ringing bell involuntarily capture attention, even if we are engaged in a completely different task. However, experimental evidence has shown that capture of attention may be reduced if not entirely eliminated under specific circumstances (Folk & Remington, 1998; Lamy et al., 2004). For many years, visual search designs have investigated these seemingly contradictory results (called the "attentional capture debate"; Luck et al., 2021). The current status of this debate centers around the proposition that attentional capture (i.e., whether or not a salient distractor produces behavioral costs when present) depends on the specific stimulus configurations used in the search arrays (Folk & Remington, 1998; Lamy et al., 2004; Luck et al., 2021).

Specifically, it was shown early on that the goal-directed search for a target composed of known features might override attentional capture by salient singleton distractors (Bacon & Egeth, 1994), i.e., stimuli that are task-irrelevant and standing out in at least one feature. In addition, there is abundant evidence that learning the irrelevant distractor’s features reduces attentional capture triggered by its presence (Cosman et al., 2018; Gaspelin et al., 2015; Gaspelin & Luck, 2018a, 2018b; van Moorselaar et al., 2021; van Moorselaar & Slagter, 2019; Vatterott et al., 2018; Weaver et al., 2017).

Another critical constraint on attentional capture was recently revealed by Wang and Theeuwes (2020), who showed that prevention of singleton capture might only occur for stimulus set sizes smaller than six. They argued that the relative salience of singleton stimuli is low for small numbers of stimuli compared to when stimulus set size is large, and that prevention of capture is the result of a serial search strategy employed in a typical four-item search paradigm. In reply, Stilwell and Gaspelin (2021) reported that singleton capture can be prevented even with higher set sizes (up to 30). They argued that the results of Wang and Theeuwes (2020) were due to floor effects due to the number of stimuli probed in the so-called "letter probe task." However, the study by Stilwell and Gaspelin (2021) and a follow-up EEG adaptation (Stilwell et al., 2022) also showed a reduced distractor presence benefit and letter probe suppression effect with increasing set sizes even when controlling the number of probed letters. Although these results, on the one hand, might suggest a reduced net benefit in suppressing one item with higher set sizes, on the other hand, they could also reflect more competition in the search for the target, as indicated by an overall reduced behavioral performance. Thus, displays of different numbers of search items cannot be compared easily. In addition, it was shown that relative stimulus saliency depends on local feature contrast and distractor homogeneity across the search display (Duncan & Humphreys, 1989; Nothdurft, 1993) and, therefore, can be high for small set sizes as well (Chang et al., 2021). Thus, whether relative stimulus saliency, even in small set sizes, is an essential factor for the prevention of capture is still a matter of dispute in the "attentional capture" debate. Knowing the role of distractor saliency would have important implications for constructing attentional models for visual search. If distractor processing is contingent on top-down control, computation of attentional priority would not necessarily include bottom-up stimulus salience (local contrast). This prediction would be consistent with the tenets of the *signal suppression hypothesis* (SSH), according to which a learned distractor with a nontarget feature value will be suppressed (Gaspelin & Luck, 2018a, 2018b; Luck et al., 2021). On the other hand, stimulus-driven salience might always play a role in visual search, but its effects may be offset by top-down inhibition; i.e., only highly salient distractor stimuli would result in net interference (Belopolsky et al., 2010; Lamy et al., 2004; Müller et al., 2009).

**Fig. 1:**
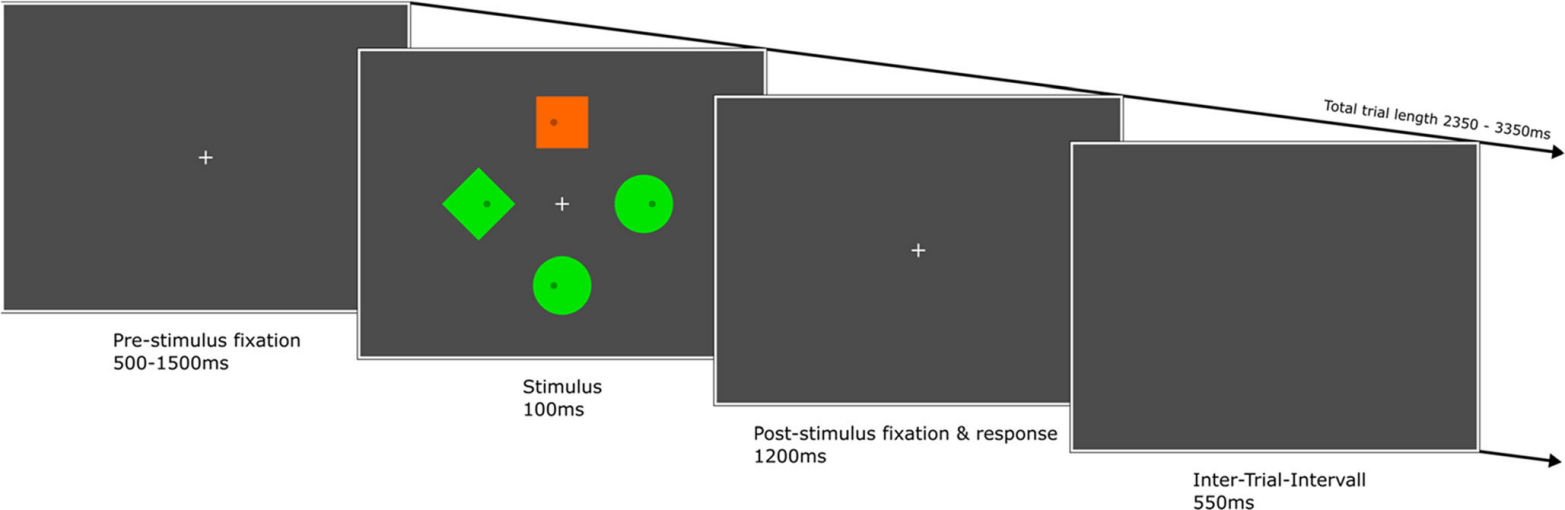
Task design. An exemplary trial started with a fixation period of 500–1,500 ms. After that, the visual search display appeared for 100 ms. Participants discriminated as fast and accurately as possible the side of the dot within the target shape. Responses were allowed throughout the post-stimulus fixation period of 1,200 ms. Finally, a blank screen marked the end of the trial. After 550 ms, the pre-stimulus fixation period of the next trial started. The target shape was either the green diamond (SD group) or the orange square (ST group). Targets and distractors could occur together or separately at randomized locations. The remaining locations were filled with green circles (fillers)

The current study investigated the effect of stimulus saliency in a typical four-item additional singleton search display and measured the electrophysiological indices of target and distractor processing in two separate groups of participants. One group discriminated the laterality of a dot within a predefined green target shape that could appear at one of four possible stimulus locations (Fig. 1), either with or without a salient task-irrelevant orange distractor at an adjacent position (Salient Distractor or SD group), while the remaining stimuli (fillers) were presented in the same color as the target. The other group (Salient Target or ST group) had the same task but with a salient orange target shape presented with green fillers with or without a green deviant distractor shape. To facilitate top-down control and the prevention of attentional capture by the singleton distractor, both target and distractor identity were kept constant for each of the groups’ participants. We expected better performance for more salient targets and explored whether a higher bottom-up salience of the distractor could offset top-down control and result in net interference, i.e., behavioral costs.

Several components of the event-related potential (ERP) have been related to the processing of target and distractor stimuli in visual search. The allocation of attention to the target stimulus is considered to be indexed by a negative event-related component with an amplitude maximum at 200–300 ms over the contralateral visual cortex, the N2pc (Berggren & Eimer, 2016; Hickey et al., 2009; Tay et al., 2019). In other studies, an earlier component, the N1pc, has also been related to attentional deployment (Ansorge et al., 2011; Eimer, 1996; Forschack et al., 2022a; Hickey et al., 2009; Hilimire et al., 2012; Kiss et al., 2008; Kiss & Eimer, 2011; Mazza et al., 2009; Verleger et al., 2012), and a recent study suggests that both components (N1pc and N2pc) are functionally equivalent (Forschack et al., 2022b).

In contrast, it is widely accepted that the prevention of attentional capture by salient distractors is reflected in the so-called Pd (distractor positivity) component that shows an increased amplitude at contralateral compared to ipsilateral parietal electrode sites (Hickey et al., 2009). The Pd is presumed to reflect the suppression of irrelevant distractor information. In support of this proposal, the Pd amplitude was reported to correlate with probe suppression in the letter probe task of the typical four-item design (Gaspelin & Luck, 2018b) and with shorter reaction times to targets (i.e., less distractor interference; Feldmann-Wüstefeld et al., 2016; Gaspar & McDonald, 2014; Sawaki et al., 2012), reduced accuracy of saccadic eye movements to salient distractors (Weaver et al., 2017) and the number of distractors in a working memory task (Feldmann-Wüstefeld & Vogel, 2019).

As mentioned above, a critical feature of search display stimuli is their saliency with respect to other items in the display. The N2pc has been reported to have a shorter latency for more salient targets (Brisson et al., 2007; Töllner et al., 2011). When it comes to distractors, one recent study that investigated distractor saliency on the Pd by increasing display set sizes failed to find any effect (Stilwell et al., 2022), but this study potentially suffers from confounding effects of distractor suppression and increased stimulus competition. In the current study, the number of items was kept the same while manipulating distractor saliency for the two groups. We aimed to investigate whether distractor saliency could offset top-down control and trigger attentional capture and whether this capture was reflected as a reduced Pd amplitude or the emergence of other attention-related components like N1pc/N2pc (see above). According to the SSH, any distractor with a nontarget feature value will be proactively suppressed to prevent attentional capture, an effect that is indexed by the emergence of the Pd (Gaspelin & Luck, 2018b; Luck et al., 2021; Stilwell et al., 2022). Thus, the SSH would predict that both low and high salient distractors will trigger the Pd in the absence of behavioral costs.

In both experimental groups, there were also trials where the distractor was presented alone with three fillers. These "distractor alone" trials were of particular interest because they allowed investigating the potential allocation of attention to the distractor in the absence of competitive interactions with the target and provided a test of the proposition that the Pd depends on the neural competition for processing resources between a task-relevant target and a salient distractor (Hilimire et al., 2012; Kiss et al., 2012). Here we tested the prediction that the Pd would not appear without such competitive interactions by additionally manipulating the saliency of the distractor/target.

Besides the Pd, alpha-band activity has also been proposed as a neural signature of stimulus suppression (Foxe & Snyder, 2011; Jensen & Mazaheri, 2010). In spatial attention studies, alpha-band amplitudes were found to be increased over the cortical hemisphere contralateral to the uncued visual field, i.e., the to-be-ignored side, relative to the cued (attended) location (Frey et al., 2014; Gould et al., 2011; Gundlach et al., 2020; Haegens et al., 2012; Kelly et al., 2006; Rihs et al., 2007; Sauseng et al., 2005; Thut et al., 2006; Worden et al., 2000; Wöstmann et al., 2016). Further, increased alpha-band amplitudes that preceded a stimulus were linked to the impairment of its perception (Al et al., 2020; Benwell et al., 2017; Busch et al., 2009; Chaumon & Busch, 2014; Forschack et al., 2020; Iemi et al., 2017; Limbach & Corballis, 2016; Mathewson et al., 2009; Romei et al., 2010; Samaha et al., 2017; van Dijk et al., 2008). Finally, post-stimulus alpha-band amplitude increases have been associated with task-irrelevant stimuli, while decreases were linked to task-relevant stimuli (Antonov et al., 2020; Gundlach et al., 2020; Payne et al., 2013; Sauseng et al., 2005; van Diepen et al., 2016; van Moorselaar & Slagter, 2019; Wöstmann et al., 2019; Zhigalov & Jensen, 2020). These changes in alpha-band activity led to the proposal that increased alpha-band amplitude (event-related synchronization; ERS) reflects perceptual inhibition (but see Foster & Awh, 2019; van Moorselaar & Slagter, 2020), whereas decreases in alpha-band amplitude (event-related desynchronization; ERD) reflect enhanced processing of stimuli (Bacigalupo & Luck, 2019; Hanslmayr et al., 2011; Klimesch, 2012). Given this extensive evidence, we recorded alpha-band oscillations in the present study as a converging neural index to evaluate the functional significance of the Pd component. If the Pd does indeed reflect stimulus suppression, we would expect concurrently recorded alpha-band amplitudes to increase over the hemisphere contralateral to the distractor either in parallel with or as a consequence of the emergence of the Pd.

## Methods

### Participants

Fifty-three normal young adults (28 female, 24 male; mean age: 23.7 years; age range: 18*–*40) participated in the experiment. Each participant was randomly assigned to one of two experimental groups: the SD (Salient Distractor) group performed the task with a non-salient target (*n* = 27) and the ST (Salient Target) group performed with a salient, color pop-out target (*n* = 26, see below for details). Participation was either compensated by class credits or financial reimbursement (10 €/h). The required sample size was calculated with a power (1-β error probability) of 0.8 and an α error probability of 0.05 using G*Power (Faul et al., 2009) based on previous findings on the effects of covert spatial attention on visual alpha oscillations (*d* ~ 0.5–0.8; Bacigalupo & Luck, 2019; Forschack et al., 2022a; Foxe & Snyder, 2011; Gundlach et al., 2020; Händel et al., 2011) and the Pd component (*d* ~ 1–2; Hickey et al., 2009; Hilimire et al., 2012). We had to exclude three participants from the final analyses because their overall number of trials was two standard deviations below the sample average and another participant because of technical problems with one channel set during the recording. Thus, 49 participants (SD group, *n* = 26; ST group, *n* = 23) remained in the final sample. All participants had normal or corrected-to-normal vision.

Before the study, participants gave written informed consent and were informed about the nature of the experiment. The study protocol followed the tenets of the Declaration of Helsinki and was approved by the local ethics committee.

### Stimuli

Stimuli were created with custom scripts using the Psychophysics toolbox 3.0.15 (Brainard, 1997; Kleiner et al., 2007) implemented in Matlab R2017b (The MathWorks, Natick, MA, USA) running in a Linux Ubuntu environment (Version 16.04, xenial). They were presented through a ProPixx DLP Projector (VPixx Technologies Inc., Canada) set to a resolution of 1,920 × 1,080 pixels and a refresh rate of 120 Hz. The projector display was a flat-screen of 63.5 × 36 cm at a 120 cm distance in front of the participant. The search array consisted of four shapes positioned symmetrically along the vertical and horizontal midline of the screen (Fig. 1). The target could be either a green diamond (i.e., a 45° rotated square, RGB 0, 1, 0) for the SD group or an orange square (RGB 1, .4, 0) of 2.4° visual angle (edge length) for the ST group. The orange square served as a salient, irrelevant distractor for the SD group and the less salient green diamond was the distractor for the ST group. Area-matched non-salient filler stimuli (circles, diameter 2.71° visual angle) were presented in green for both groups. On some trials, the target or distractor was presented with three non-salient fillers only (target/distractor-alone conditions) and on other trials both target and distractor were presented together with two non-salient fillers (target/distractor-competition condition). Thus, the SD group searched for a non-salient target, while the ST group searched for a salient singleton color pop-out target. It should be emphasized that the physical stimuli were identical for the two groups; only the target and distractor designations were swapped. When distractor and target were presented together, they always appeared at a vertical and adjacent horizontal position and never in the opposite positions (i.e., left/right or upper/lower). Centers of the stimuli were located at 4.2° of visual angle from the screen center, on which a white (RGB .8, .8, .8) fixation cross with a bar length of 0.24° and a bar width of 0.05° of visual angle was presented throughout the trial. All colors underwent individual isoluminance adjustments with a grey background (RGB .1, .1, .1) as reference (approximately 60 cd/m^2^) utilizing heterochromatic flicker photometry (Wagner & Boynton, 1972). The background during stimulus presentation was a darker grey (RGB .05, .05, .05; approximately 30 cd/m^2^). On all trials, all stimuli contained a dot (diameter of 0.26°) randomly presented at 0.4° of visual angle either to the right or the left from the stimulus center.

### Experimental procedure and task

Overall, there were nine stimulation conditions. In one condition, only the four green circles were presented. That condition was considered a "baseline measure," in addition to the pre-stimulus baseline, to allow an evaluation of post-stimulus alpha-band amplitudes representing either stimulus facilitation or inhibition (Schneider et al., 2022; for further details see the Online Supplementary Material (OSM)). In the remaining eight conditions, the target or the distractor singleton appeared at random at either the left or the right positions and either together with or without a vertically presented distractor singleton or target (top or bottom position, equally distributed across trials), respectively. We instructed participants to search for the target shape (green diamond or orange square, depending on group) and indicate at which side (left or right) the target dot appeared on every trial by pressing the left or right arrow buttons of the keyboard. Each trial started with a fixation period where only the fixation cross was visible for 500 to 1,500 ms, after which all four shapes appeared simultaneously for 100 ms, followed by a post-stimulus fixation interval of 1,200 ms, in which responses were recorded, and an inter-trial interval of 550 ms. Thus, the mean trial length was 2.85 s (2.35*–*3.35 s). After that, the pre-stimulus fixation period of the next trial started.

Before the actual experiment started, participants were trained on the task. During training, dot luminance varied on every training trial from .01 to .05 RGB values (five steps) below the individually adjusted stimulus luminance weighted by its maximum RGB value. One training run consisted of 50 trials, after which a Weibull function was used to model the participants' response rates (percentage correct) at the dot luminance values (Kingdom & Prins, 2010) and was repeated until a proper fit was reached (usually not more than twice).

To avoid ceiling effects and to homogenize the sample regarding behavioral performance, dot luminance was varied around the modeled point estimate of 85% correct responses by ± 0.005 RGB values (i.e., three luminance steps) during the experiment. Overall, there were 18 blocks with 50 trials per block, resulting in 100 randomly presented trials for each of the nine conditions. Blocks were separated by short, participant-paced breaks. Participants responded with either the left or the right hand during the first nine blocks, after which the responding hand was switched (starting hand randomly assigned across participants).

### Experimental conditions for statistical analysis

We pooled trials of comparable experimental conditions as follows: (1) All trials in which the target shape was left or right, and the singleton distractor was at the top or the bottom position. We refer to this condition as *target lateral – distractor vertical* (or TLDV). (2) All trials in which the target was at the top or bottom position and the singleton distractor was either left or right were pooled to form the condition *distractor lateral – target vertical* (or DLTV). Trials containing only a target at the left or right position were also pooled and called *target lateral* (or TL). Finally, trials without a target but with a distractor at the left or right position were pooled together as distractor lateral (or DL) trials.

### Behavioral analysis

Trials in which responses were faster than 400 ms or slower than 1,000 ms were excluded from analyses. To test the effect of distractor/target saliency (orange square or green diamond) on behavioral performance, proportion correct discrimination performance and reaction times were modeled by linear mixed-effects models (lme4 package in R) with the factors "condition" (TLDV, DLTV, TL) and "sub-group." Factor combinations were statistically assessed by log-likelihood ratio tests. Pairwise group comparisons (SD vs. ST group) for each condition were achieved by a two-sample t-test assuming equal variance and Bayes factor tests to assess the odds ratio of null and alternative hypotheses employing the standard JZS prior with a scaling factor r = √2/2 ≈ 0.707 (Forschack et al., 2020; Rouder et al., 2009). Correction for multiple comparisons was achieved by false discovery rate if required (Benjamini & Hochberg, 1995).

### Electrophysiological recording

EEG was recorded from 64 Ag/AgCl electrodes mounted in an elastic cap with an ActiveTwo Amplifier (BioSemi) at a sampling rate of 512 Hz, employing an anti-aliasing low-pass filter of 104 Hz stored for later offline analysis. Two electrodes were placed horizontally at the canthi of both eyes and vertically above and below the right eye to measure horizontal and vertical eye movements and blinks.

### General preprocessing of electrophysiological data

For offline data analysis, the EEGLAB toolbox (Delorme & Makeig, 2004) and custom MATLAB scripts (The MathWorks) were used. In a first step, we ran the standardized early-stage EEG processing pipeline (PREP v0.55.3; Bigdely-Shamlo et al., 2015) on the continuous data. The algorithm re-referenced the continuous data to a robust average reference signal derived by iteratively detecting and interpolating noisy channels (interpolation based on all but the VEOG and HEOG electrodes). Next, individual datasets underwent independent component analysis (ICA; adaptive mixture of independent component analyzers (AMICA); Palmer et al., 2011) to identify sources of ocular and muscle artifacts as well as signals of other non-neural origins (Delorme et al., 2012; Li et al., 2006; Pion-Tonachini et al., 2019). Before ICA, datasets were prepared by applying the following procedures: training datasets for ICA were high-pass filtered with 1 Hz, all blocks were concatenated, and contiguous epochs of 1 s were extracted, from which the average epoch potential was subtracted. These epochs were then screened for non-stereotypical artifacts and rejected if contaminated. After ICA, the "ICLabel" classifier (v1.0.1) computed IC class probabilities across seven classes (Brain, Muscle, Eye, Heart, Line Noise, Channel Noise, Other) based on the labels of an artificial neural network that was trained on expert crowd labeled IC datasets (Pion-Tonachini et al., 2019). Only the unmixing and sphering matrices of components that were classified as "Brain" or "Other" components by a probability of at least 0.42 were forward-projected to the continuous dataset that was subsequently high- and low-pass filtered for further analyses (function "pop_firws" v2.1, Widmann et al., 2015; 1. step high-pass: low cut-off of 0.5 Hz, Kaiser window, maximum passband deviation: 0.001 and transition bandwidth: 1 Hz, resulting in filter order/ length of 1856 data points; 2. step low-pass: high cut-off of 17 Hz , Kaiser window, maximum passband deviation of 0.0001 and transition bandwidth of 4.25 Hz, resulting filter order/ length of 606 data points estimated by the *pop_firwsord* function). On average, 23 (5 SD) out of 58 (4 SD) components were rejected. The average class probability of rejected, “Brain,” and “Other” components was 0.65 (0.07 SD), 0.80 (0.05 SD), and 0.6 (0.05 SD), the average percentage of data variance they accounted for was 61.8% (20.9% SD), 35.2% (20% SD), and 3.2% (2.3% SD), respectively. Proper epochs were extracted from the continuous channel signals ranging from -1,000 to 1,000ms relative to stimulus onset (t = 0), from which the individual epoch mean was subtracted. Epochs exceeding an adaptive channel threshold for blinks and eye movements exceeding a potential threshold of 30 μV (Berggren & Eimer, 2018) within -350 to 350 ms were discarded after manually reviewing the alleged artifactual epochs. The following average of trials per condition across both experiments remained for statistical analyses: 174 (20 SD) TLDV, 175 (17 SD) DLTV, 175 (21 SD) TL, and 170 (15 SD) DL.

As a final preprocessing step, data were transformed to reference-free current source densities (CSDs) by computing the surface Laplacian to focus on high spatial frequency components like N2pc, Pd, and alpha, to reduce volume-conducted potentials or distributed sources (Perrin et al., 1989).

### Analysis of event-related potentials

**Fig. 2:**
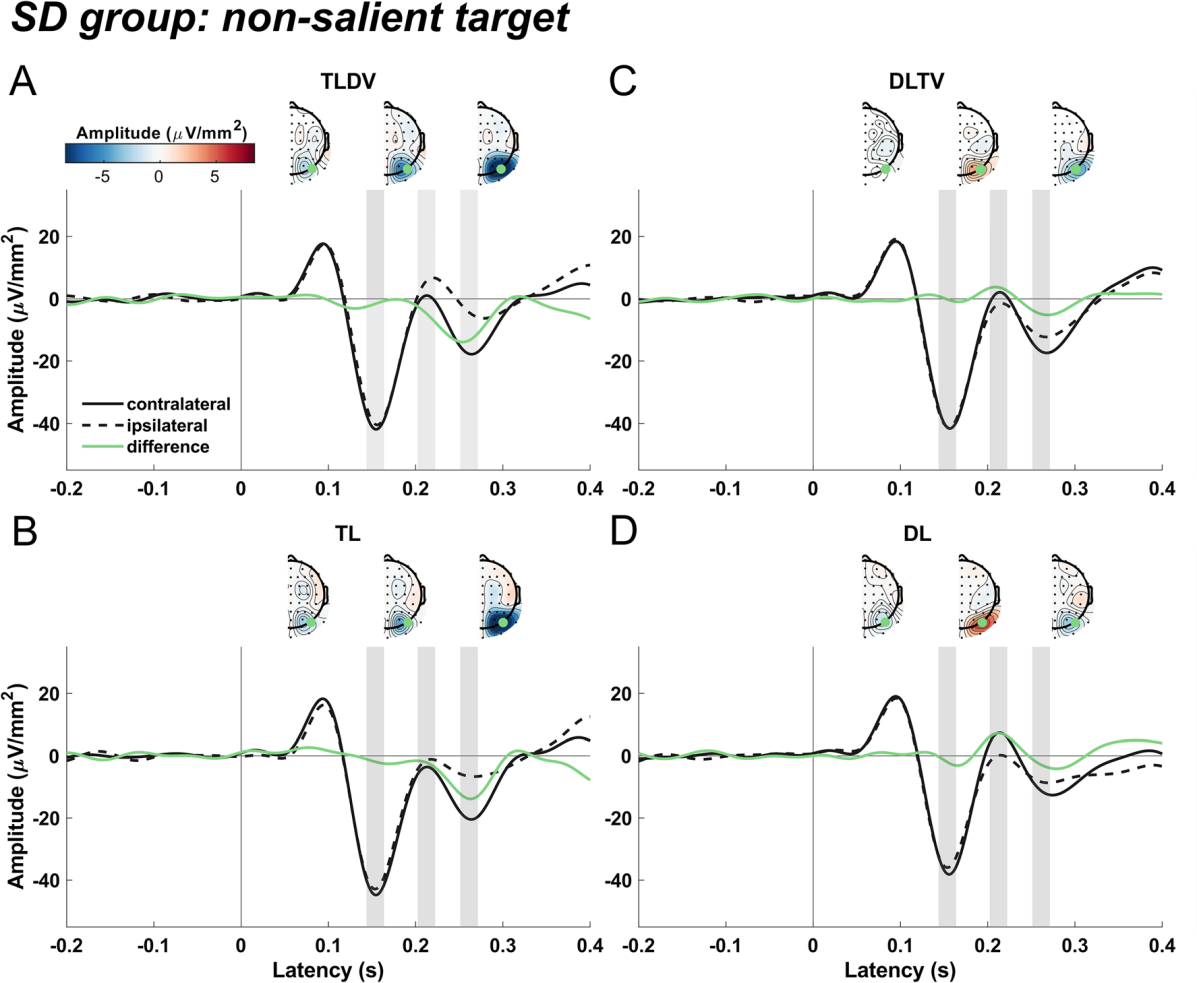
Event-related current source densities contra- and ipsilateral to the non-salient green diamond target and salient orange square distractor extracted at P08 and PO7 for the conditions "target lateral - distractor vertical" (**a**, TLDV), "single target lateral" (**b**, TL), "distractor lateral - target vertical" (**c**, DLTV) and "single distractor lateral" (**d**, DL). The difference potential between contra- and ipsilateral scalp sites is given in orange for each condition. Zero marks the onset of the visual search display. Grey shaded areas indicate the three time windows used for averaging current source density (CSD) values of the lateralized potential for further statistical analysis (window 1: 144–164 ms, window 2: 202–222 ms, window 3: 251–271 ms). Insets reflect the topographical CSD distribution of the contralateral minus ipsilateral difference collapsed across left and right hemispherical electrodes (see Methods section for details) and averaged for each of the three windows

Artifact-free trials were averaged for each participant and experimental condition. We pooled across left versus right lateral stimulus presentations to extract ERP components and calculated the difference ERPs (contralateral minus ipsilateral) at electrodes PO7/PO8 (Gaspelin & Luck, 2018b). These difference ERPs were baseline corrected by the mean voltage between -200 and 0 ms relative to stimulus onset. CSD values of difference ERPs were averaged within three fixed time windows of equal length to encompass the peak amplitudes of the N1pc, N2pc, and Pd components for all conditions and both groups to test for lateralized potentials and track possible latency differences between groups. For this, ERPs contra- and ipsilateral to target and distractor stimuli were averaged separately across participants, and the analysis windows were centered at the grand-average N1 component and the two subsequent peaks (see OSM Fig. 2). Additionally, latency differences between the groups were tested by running t-tests for each group and pooled target lateral or distractor vertical conditions, respectively. Threshold-free cluster enhancement (TFCE) in the time domain with a cluster threshold of p = 0.05 (cluster size exponent E = 0.5, statistical intensity exponent H = 2; Forschack et al., 2020; Mensen & Khatami, 2013; Smith & Nichols, 2009) and 100,000 permutations, identified (multiple comparisons corrected) clusters of significant deviation from baseline for each group and condition (target lateral and distractor lateral trials pooled, respectively). Additional analyses quantified the difference in the lateralized potential amplitudes between the TLDV-TL and DLTV-DL conditions. We anticipated a larger Pd in the competition condition (DLTV) than in the single distractor condition (DL, Hilimire et al., 2012; Kiss et al., 2012). If top-down distractor rejection is offset (counteracted or diminished) by increasing the saliency of the distractor, we expected the Pd to be present in the ST group searching for the salient target but diminished or absent in the SD group searching for the non-salient target, because in the latter case the salient distractor captures attention (Lamy et al., 2004). If, however, distractor processing is purely contingent on top-down guidance of attention towards the target, there should not be any effect of distractor saliency on the Pd (Luck et al., 2021; Stilwell et al., 2022).

### Analysis of alpha amplitude time courses

To extract alpha-band activity, every trial was convolved with Gabor kernels centered at 9*–*12 Hz (steps of 0.5 Hz, ± 1.4 Hz FWHM) in the frequency domain and subsequently averaged across trials and frequencies. Alpha current source density values (αCSDs) for the time window from -500 to 800 ms were extracted from a broader cluster of electrodes, including electrodes for the ERP analysis (right cluster: PO8, PO4, O2, P10, P8; left cluster: PO7, PO3, O1, P9, P7; Bacigalupo & Luck, 2019; Forschack et al., 2022a) contralateral and ipsilateral to the laterally presented target or distractor from which the baseline period (-500 to -200 ms relative to search display onset) was subtracted. As in our previous study (Forschack et al., 2022a), a time window from 400 to 800 ms, reflecting the start of hemispheric lateralization after an initial alpha suppression, was identified. Averaged αCSD values in that time window were submitted to a within-subject repeated-measures ANOVA with the factors *laterality* (contra vs. ipsi) *x condition* (TLDV, DLTV, TL, DL) x *group* (SD vs. ST). Planned post hoc comparisons were achieved by paired t-tests and correction for multiple comparisons by false-discovery rate (Benjamini & Hochberg, 1995) if necessary. With alpha-band lateralization as a marker of attentional deployment in space, we would expect increased values at contralateral relative to ipsilateral electrode sites for the search conditions with a lateral distractor if it can be suppressed. If top-down distractor rejection is offset by increasing the saliency of the distractor (Lamy et al., 2004), we expected increased contralateral relative to ipsilateral alpha-band amplitudes in the ST group searching for the salient target but a reduced lateralization in the SD group searching for the non-salient target, because the salient distractor in that group would capture attention. If, however, distractor processing is purely contingent on top-down guidance of attention towards the target, there should not be any effect of distractor saliency on alpha lateralization.

## Results

### Behavior

Comparisons between the salient distractor (SD) and the salient target (ST) groups did not reveal any significant differences regarding correct responses (all *t* < 1.5 and < 2, see Table 1), which was expected as both groups were trained to achieve ~85% correct discriminations. However, reaction times were generally faster in the ST group in all conditions, which is confirmed by Bayes factors showing up to 3.1 times more evidence in favor of the alternative hypothesis in the condition with the lowest behavioral performance (i.e., DLTV, see Table 2). Thus, a more salient target speeds up reaction times.

**Table 1:** Group averages of behavioral performance during the discrimination task with standard deviation (STD), group range for the main experimental conditions, and statistical values of condition-wise group comparisons

Group	Condition	Mean (%)	STD (%)	Range (%)	df	t-value	p-value	Cohens *d*	BF_01_
SD	TLDV	88.8	7.2	70–97	47	1.05	.3	0.3	2.2
ST		86.7	6.5	75–98					
SD	TL	88.7	8.1	63–97	47	0.84	.4	0.24	2.6
ST		86.9	6.9	75–98					
SD	DLTV	79.7	12.7	52–99	47	1.4	.16	0.41	1.6
ST		74.7	11.4	49–97					

TLDV target lateral, distractor vertical, DLTV distractor lateral, target vertical, TL target lateral only, *SD group* green diamond target, salient orange square singleton, *ST group* orange square target, non-salient green diamond singleton

Bayes factor BF_01_ indicates evidence in favor of the null hypothesis

**Table 2:** Group-specific average reaction times during the discrimination task with standard deviation (STD), group range for the main experimental conditions, and statistical values of condition-wise group comparisons

Group	Condition	Mean (ms)	STD (ms)	Range (ms)	df	t-value	p-value	Cohens *d*	BF_10_
SD	TLDV	612	54	520–745	47	2.4	**.02**	0.67	2.6
ST		575	57	478–741					
SD	TL	606	50	517–738	47	2.1	**.04**	0.6	1.7
ST		574	58	479–731					
SD	DLTV	641	59	560–776	47	2.45	**.018**	0.7	3.1
ST		600	59	489–777					

*TLDV* target lateral, distractor vertical, *DLTV* distractor lateral, target vertical, *TL* target lateral only*, SD group* non-salient green diamond target, salient orange square singleton*, ST group* salient orange square target, non-salient green diamond singleton

T-values were calculated with two-sided independent-samples tests between the SD and ST group for each condition. *p*-values signifiant at *p* < 0.05 are given in bold. Bayes factor BF_10_ indicates evidence in favor of the alternative hypothesis, i.e., that the groups differ

Testing the factors of the linear-mixed effects models confirmed this picture as the main effect of “group” was only significant regarding reaction times (*χ*^*2*^(1) = 5.57, *p* = 0.018) but not correct responses (*χ*^*2*^(1) = 1.82, *p* = 0.18; see Table 3). Furthermore, the main effect of “condition” was significant for both performance measures (correct responses: *χ*^*2*^(1) = 66.45, *p* < 0.001; reaction time: *χ*^*2*^(1) = 62.1, *p* < 0.001).

**Table 3:** Factor tests after modeling the relationship between behavioral performance and the factors "group" and "condition." Test statistics indicate log-likelihood ratio tests with the model of the previous row. Thus, degrees of freedom (df) and chi-square reflect difference tests of two models. *p*-values significant at *p* < 0.05 are given in bold

Factor	Response	Log-likelihood	df	*χ*^*2*^	p-value
Intercept only	Proportion correct	131.59			
	Reaction times	271.74			
Group	Proportion correct	132.50	1	1.82	.18
	Reaction times	274.52	1	5.57	**.018**
Condition	Proportion correct	165.72	1	66.45	**<.001**
	Reaction times	305.58	1	62.12	**<.001**
Group + condition	Proportion correct	166.63	1	1.82	.18
	Reaction times	308.37	1	5.57	**.018**
Group*condition	Proportion correct	167.64	2	2.01	.37
	Reaction times	309.31	2	1.89	.39

To further investigate the effect of condition in each group, we performed paired t-tests. For both groups, these tests revealed that in competition trials (target with distractor), participants were better in discriminating the side of the dot if the target shape appeared at the lateral position compared to when the target appeared vertically (see Table 1; SD group : *t*(25) = 4.7; *p* < 0.001, *d* = 0.88; ST group : *t*(22) = 5.4; *p* < 0.001, *d* = 1.28). Furthermore, reaction times were *faster* in the TLDV compared to the DLTV condition for both groups (SD group: *t*(25) = -4.7; *p* < 0.001, *d* = 0.51; ST group : *t*(22) = -4.6; *p* < 0.001, *d* = 0.43; Table 2). Similar to these effects, behavioral performance differed between the TL and the DLTV conditions regarding correct responses (SD group: *t*(25) = 4.5; *p* < 0.001, *d* = 0.85; ST group : *t*(22) = 5.5; *p* < 0.001, *d* = 1.29; Table 1) and reaction times (Table 2; SD group : *t*(25) = -5.6, *p* < 0.001, *d* = 0.64; ST group: *t*(22) = -5.2; *p* < 0.001, *d* = 0.44).

Given these differences in behavioral responses between target positions at the vertical or horizontal midline, in the following, we only analyzed reaction time data when the target was at a lateral position to test for possible attentional capture effects instead of pooling across all target positions. This is also motivated by the fact that in "target alone" conditions, the target appeared either left or right only.

Importantly, comparison of distractor present versus absent conditions (when the target was presented laterally) will indicate whether or not the distractor produced perceptual costs. For both groups, proportion of correct responses was not significantly different between TLDV and TL conditions (SD group: *t*(25) = 0.09; *p* = 0.93, *d* = 0.01; ST group: *t*(22) = -0.3; *p* = 0.77, *d* = 0.03), which was supported by 4.8 and 4.4 times more evidence for the null hypothesis, respectively. Reaction times, however, were significantly faster when the salient distractor was absent in the SD group: *t*(25) = 2.9; *p* = 0.008, *d* = 0.11), but there was no difference when a non-salient distractor was absent in the ST group: *t*(22) = 0.5; *p* = 0.65, *d* = 0.01, *BF*_*01*_ = 4.1, see also OSM Fig. 1).

### Event-related potentials

#### SD group: Searching for a green diamond

Figure 2 shows ERPs extracted from PO8 and PO7 sites contra- and ipsilateral to target and singleton distractor shapes for the SD group that searched for the non-salient green diamond. For the "target lateral "conditions (TLDV and TL; Fig. 2a and b), we expected an N2pc, while for the "distractor lateral – target vertical" condition (DLTV; Fig. 2c), we expected a Pd as reported in the study by Gaspelin and Luck (2018b, Experiment 2).

With respect to the N2pc, our expectations were confirmed by the results of the third time window between 251 and 271 ms after stimulus onset (TLDV: *t*(25) = -6.5, *p* < 0.0001; TL: *t*(25) = -6.1, *p* < 0.0001 with no difference in N2pc amplitude between TLDV and TL: *t*(25) = 0.28, *p* = 0.78, *d* = 0.03, *BF*_*01*_ = 4.7). The Pd emerging in the second time window (202–222 ms, DLTV: *t*(25) = 3.3, *p* = 0.003) was consistent with our predictions. Also, consistent with a previous study by Gaspelin and Luck (2018c), the Pd to the distractor was followed by a lateralized negativity in the time window of the N2pc (window 3, Fig. 2c; *t*(25) = -4.2, *p* < 0.001).

In addition, there was a significant lateralized positivity in the time window of the Pd (window 2) when the salient singleton distractor was presented alone (Fig. 2d)*,* DL: *t*(25) = 7.4, *p* < 0.0001). Contrary to our hypothesis, the Pd was smaller when the lateral distractor appeared together with a vertical target compared to when only the distractor was presented (DLTV vs. DL: *t*(25) = -3.4, *p* = 0.002, *d* = 0.66; the subsequent negativity was not significant, *t*(25) = -1.7).

In the first time window (144–164 ms), the large N1 component was significantly larger over the contralateral hemisphere for the non-salient target presented laterally together with a salient distractor at a vertical position, reflecting the emergence of the N1pc (*t*(25) = -2.24, *p* = 0.034). This lateral asymmetry of the N1 was absent when the salient distractor was presented laterally and the target appeared at a vertical position (*t*(25) = -0.1, *p* = 0.9, *BF*_*01*_ = 4.8), while it just missed significance for the TL (*t*(25) = -1.98, *p* = 0.059) and the DL (*t*(25) = -2, *p* = 0.056) conditions.

#### ST group: Searching for an orange square

**Fig. 3:**
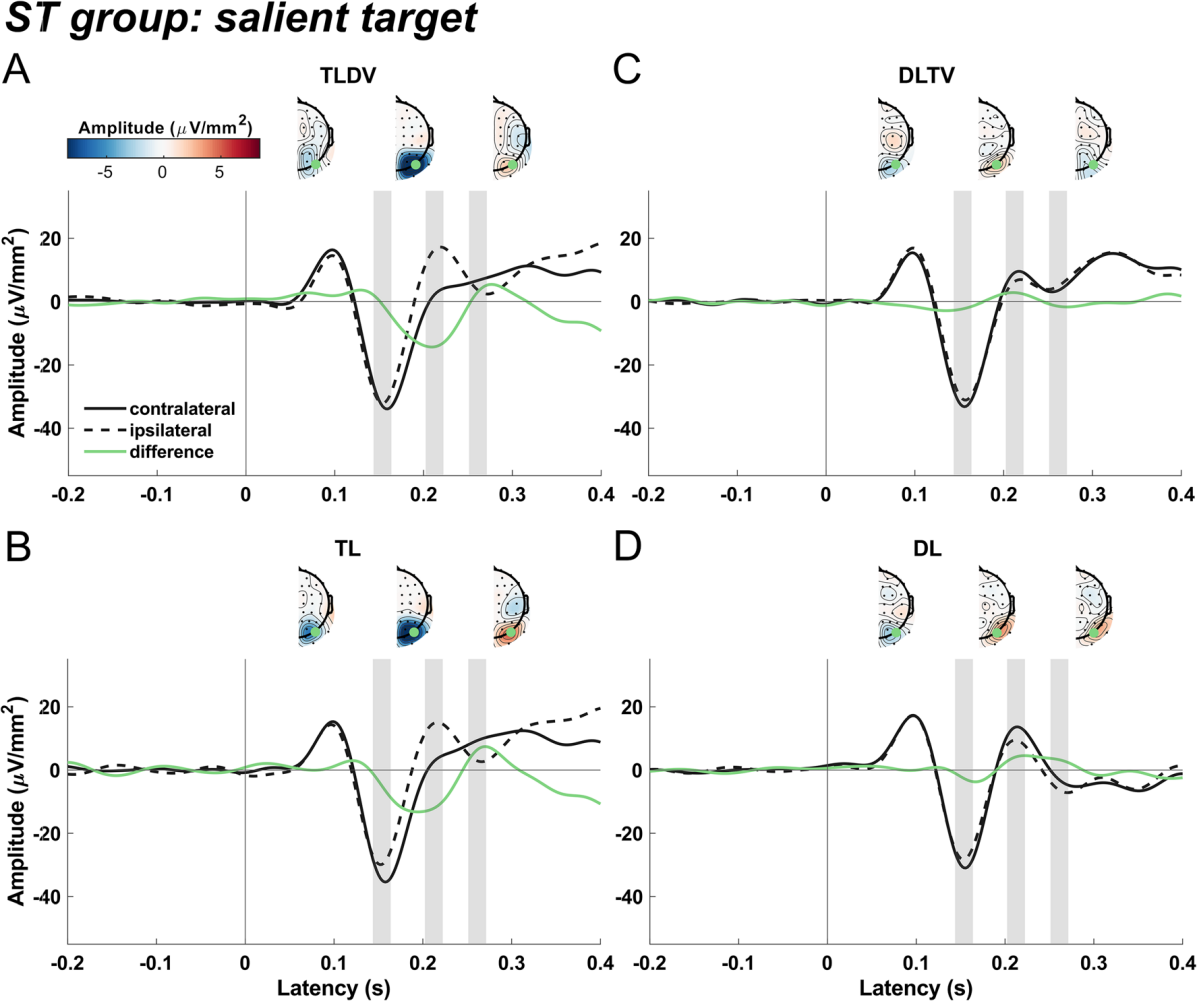
Event-related current source densities extracted at P07 and P08 sites contralateral and ipsilateral to the stimulus and topographic current source density (CSD) distributions of the contralateral minus ipsilateral difference for the ST group that received the salient orange square target and non-salient green diamond distractor. Other figure conventions are identical to those of Fig. 2

Figure 3 shows ERPs extracted from PO8 and PO7 sites contra- and ipsilateral to target and distractor shapes for the ST group that searched for the salient orange square.

Results revealed an N2pc in both target lateral conditions, which peaked about 50 ms earlier than the N2pc elicited in the SD group, resulting in a significant peak in time window 2 for both, the TLDV (*t*(22) = -6.7, *p* < 0.0001) and the TL condition (*t*(22) = -5.4, *p* < 0.0001). N2pc amplitude of TLDV was more negative than for TL: *t*(22) = -2.2, *p* = 0.037, *d* = 0.2), however, the N2pc of TL (*t*(22) = -4.9, *p* = 0.0001) but not TLDV (*t*(22) = -1, *p* = 0.34) already commenced in window 1, evident as a significant difference between the two conditions in that window (TLDV vs. TL: *t*(22) = 4.3, *p* < 0.001, *d* = 0.76). When the salient target was presented laterally without a non-salient distractor, this N2pc was followed by a positivity in the third time window (*t*(22) = 2.5, *p* < 0.02; TLDV vs. TL: *t*(22) = -3.48, *p* = 0.002, *d* = 0.29).

Both distractor lateral conditions evoked a Pd in window 2 (DLTV: *t*(22) = 4.99, *p* = 0.0001; DL: *t*(22) = 3.9, *p* < 0.001) that were preceded by a lateralized N1pc in window 1 (DLTV: *t*(22) = -2.4, *p* = 0.027; DL: *t*(22) = -2.7, *p* = 0.012). For the non-salient distractor presented alone, the Pd extended until window 3 (*t*(22) = 3.6, *p* < 0.002), resulting in a significant difference between DLTV and DL in that window (*t*(22) = -3.18, *p =* 0.004, *d* = 1.07).

#### Comparison between SD and ST groups

Obviously, there were marked differences in the ERP pattern between the two groups. First, while the SD group only showed an N2pc after search display onset in the target lateral conditions, in the ST group the N2pc was followed by a positive-going potential that, however, was only significant in the TL condition. Second, the Pd after lateral distractor presentations was significant in both groups but larger in the DL compared to DLTV only for the SD group. Conversely, in the DLTV but not the DL condition, there was a late negativity in window 3. Additionally, an early negative-going difference wave (N1pc) roughly peaking at 150 ms preceded the Pd in the distractor lateral alone condition of the ST group, suggesting an initial attentional capture of the non-salient shape singleton distractor.

**Fig. 4:**
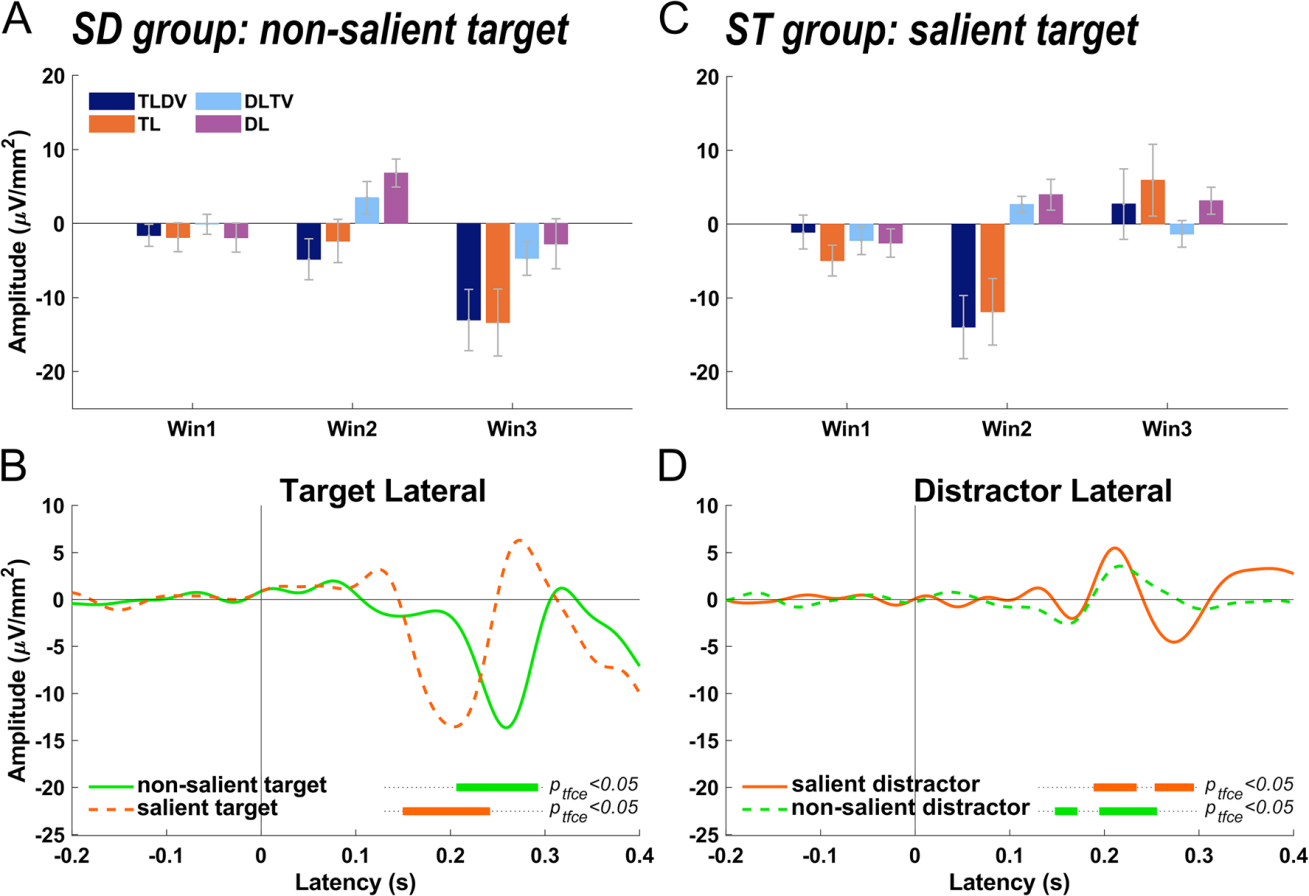
(**a+c**) Grand mean lateralized current source density values averaged for each group and analysis window (Win1: 144–164 ms, Win2: 202–222 ms, Win3: 251–271 ms) for conditions TLDV, TL, DLTV, and DL. Error bars indicate the 95% confidence interval of a t-test against zero. Superposition of ERP difference waves for the two different groups and averaged across both target lateral (**b**) and both distractor lateral (**d**) conditions, respectively. Line colors reflect target/distractor colors for the two groups. Difference waves are tested against zero in the time range from 130 to 300 ms relative to stimulus onset. The bold horizontal lines indicate significant lateralized potentials. The color indicates group identity. Zero marks the onset of the visual search display. p_tfce_<0.05; p-value threshold after correction for multiple comparisons*.* TLDV *target lateral, distractor vertical*, DLTV *distractor lateral, target vertical,* TL *target lateral only, DL distractor lateral*

Third, as indicated above, there was a significant latency difference of the N2pc but not the Pd between the two groups. To illustrate this effect, Fig. 4b and d show an overlay of the difference waves averaged across both target lateral (TLDV and TL) and both distractor lateral (DLTV and DL) conditions for the two groups, respectively. A permutation test revealed a significant N2pc cluster that started 56 ms earlier for the ST group than for the SD group. In the distractor lateral conditions, the Pd onset latencies differed only by 6 ms between the groups.

To further investigate the effect of stimulus saliency, overall amplitude differences of N2pc and Pd components between groups were tested based on the designated electrode clusters. For the ST group, the N2pc was computed as the window average ranging from 154 to 204 ms and 199–218 ms for the Pd. For the SD group, 210–260 ms window averages were used for the N2pc and 193–212 ms for the Pd. However, these tests did not reveal any significant amplitude differences between the groups (N2pc: *t*(47) = -0.06, *p* = 0.95, *d* = 0.02, *BF*_*01*_ = 3.5; Pd: *t*(47) = 1.2, *p* = 0.25, *d* = 0.34, *BF*_*01*_ = 1.99).

### Posterior alpha-band activity

**Fig. 5:**
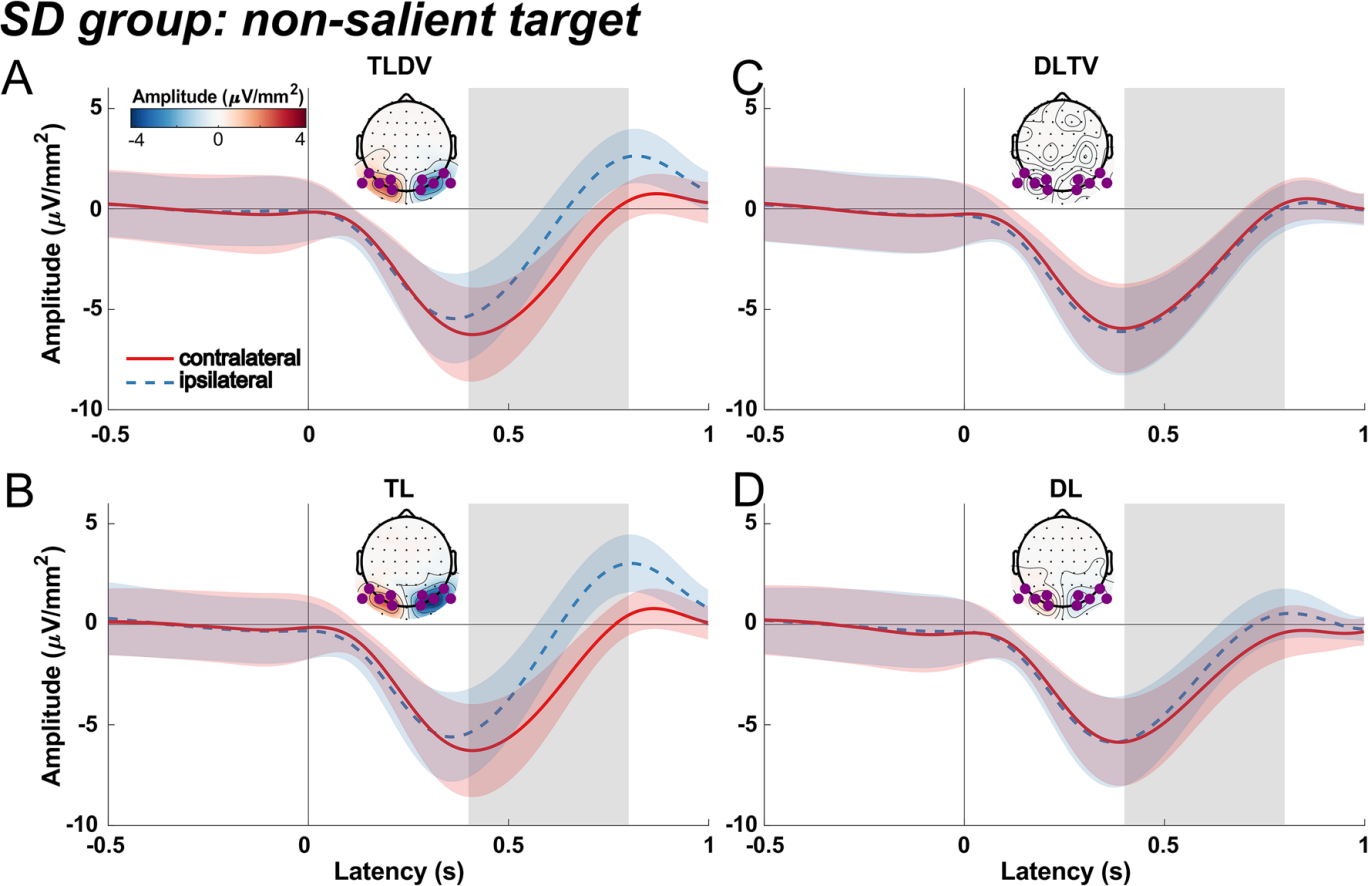
Contra- and ipsilateral alpha source current density time courses for the SD group with a non-salient target and a salient singleton distractor, corrected by a mean prestimulus baseline between -500 and -200 ms relative to search display onset. Mean values were extracted at the symmetrical electrode clusters indicated by the purple dots of the topographical insets for the conditions "target lateral - distractor vertical" (**a**, TLDV), "single target lateral" (**b**, TL), "distractor lateral - target vertical" (**c**, DLTV) and "single distractor lateral" (**d**, DL). Topographical distributions show the difference between left and right target or distractor presentations, respectively. Colored shaded areas show within-subject confidence intervals. The difference between contralateral and ipsilateral amplitudes was compared in the time range from 400 to 800 ms relative to stimulus onset indicated by the grey boxes. Zero marks the onset of the visual search display

**Fig. 6:**
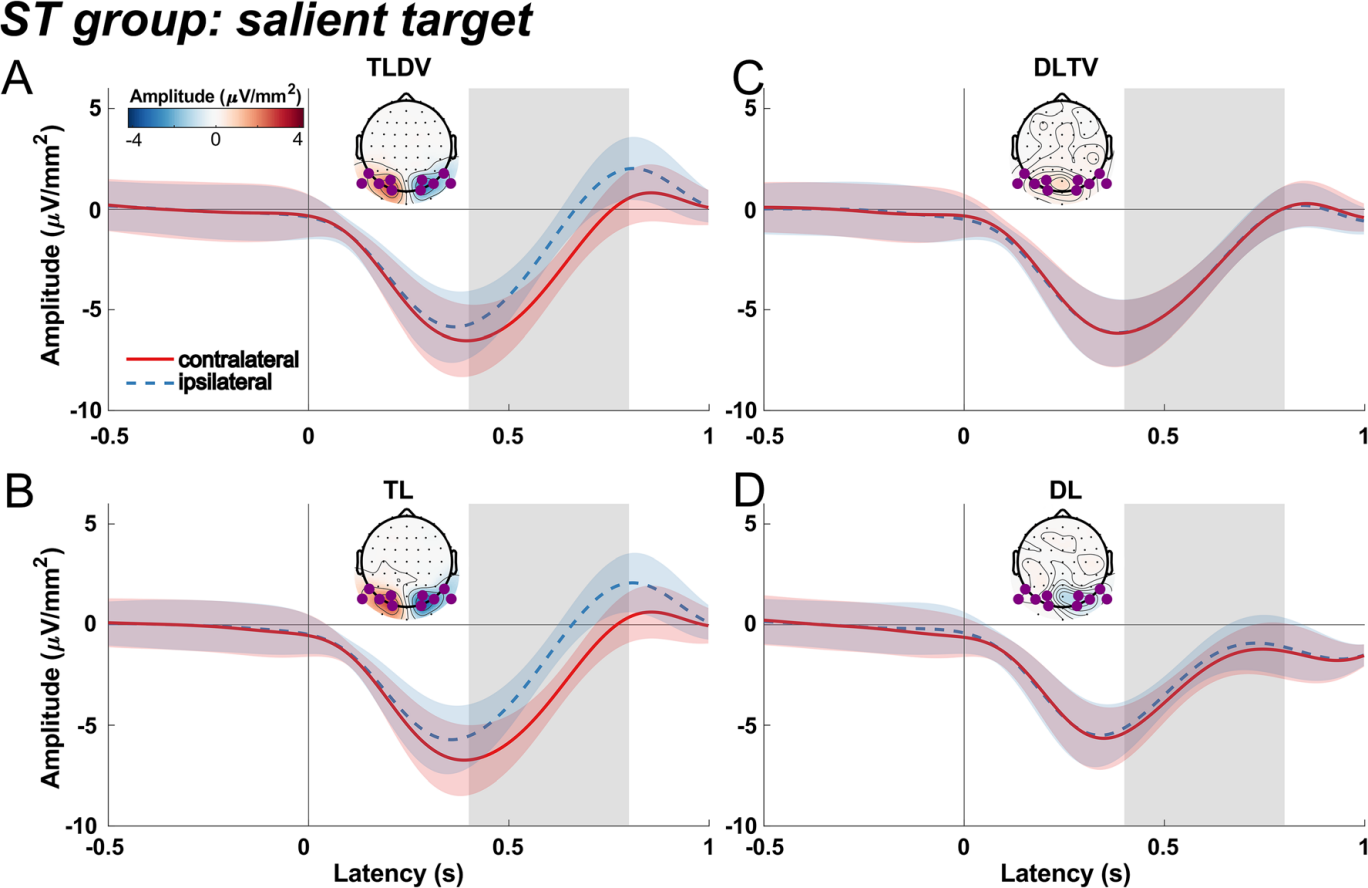
Contra- and ipsilateral alpha source current density time courses for the ST group with a salient target and a non-salient singleton distractor. Other figure conventions are the same as those in Fig. 5

As can be seen in the alpha-band time courses and the corresponding 95% within-subject confidence intervals (according to (Cousineau, 2005; Morey, 2008) in Figs. 5 and 6, both groups exhibited a significant event-related desynchronization (ERD) after the onset of the visual search display, regardless of whether a target or a distractor appeared at a lateral position.

We expected target processing to be reflected in a relative decrease of contralateral compared to ipsilateral alpha amplitude, and distractor processing (if it involved suppression) in a relative increase of contralateral alpha for both experimental groups, but stronger in the ST than the SD group. To test these predictions, the maximum linear mixed-effects model of contralateral and ipsilateral αCSD values averaged from 400–800 ms relative to search display onset were submitted to a step-by-step backward elimination procedure to obtain significant factors. Results of this procedure are reported in Table 4. As can be seen, there was a significant *laterality x condition* interaction, as well as a significant main effect of laterality (*F*(1,336) = 70.1, *p* < 0.001) and condition (*F*(3,336) = 8.6, *p* < 0.001). Interestingly, there was no difference between the two groups in alpha lateralization.

**Table 4:** Results of backward elimination procedure to model alpha ERD with the factors laterality (contra, ipsi), condition (TLDV, TL, DLTV, DL), and group (SD, ST). The factor that cannot be eliminated from the model without significantly decreasing model fit is given in bold. Winning model was Alpha ~ Laterality + Condition + Laterality x Condition + (1 | Subj). Degrees of freedom correspond to the numerator and denominator df estimated via the Kenward-Roger approximation

Factor	Eliminated	df	*F-*value	*p-*value
LATERALITY x CONDITION x GROUP	Yes	(3, 329)	0.33	0.8
CONDITION x GROUP	Yes	(3, 332)	1.07	0.36
LATERALITY x GROUP	Yes	(1, 335)	0.99	0.32
GROUP	Yes	(1, 47)	0.002	0.96
*LATERALITY x CONDITION*	No	(3, 336)	16.1	**<0.001**

*ERD* event-related desynchronization, *TLDV* target lateral, distractor vertical, *DLTV* distractor lateral, target vertical, *TL* target lateral only*, DL* distractor lateral, *SD group* non-salient green diamond target, salient orange square singleton distractor*, ST group* salient orange square target, non-salient green diamond singleton

FDR-corrected planned post hoc comparisons revealed significant differences between contra versus ipsilateral electrodes in both target lateral conditions (TLDV: *t*(48) = -7.1, *p*_*fdr*_ < 0.001, *d* = 0.28, *BF*_*10*_ = 2147300; TL: *t*(48) = -8.6, *p*_*fdr*_ < 0.001, *d* = 0.34, *BF*_*10*_ = 393650000) and the distractor alone condition (DL: *t*(48) = -3.8, *p*_*fdr*_ = 0.001, *d* = 0.08, *BF*_*10*_ = 59.8). The distractor lateral – target vertical condition was not significant (DLTV: *t*(48) = 0.76, *p*_*fdr*_ = 0.48, *d* = 0.01, *BF*_*01*_ = 4.9), thus accounting for the *laterality x condition* interaction.

To further qualify this interaction effect, pairwise comparisons were computed for all condition combinations and separately for contra- and ipsilateral electrode sites. Results are given in Table 5*.* As can be seen, the ipsi- but not the contralateral alpha ERD differed between conditions. Therefore, the absent laterality effect in the DLTV condition seems to originate from a relatively larger ipsilateral ERD in that condition that is similar to the contralateral ERD of the same and other conditions (see also OSM Analysis*,* especially OSM Figs. 3 and 4 for an attempt to control potential bilateral signal variance).

**Table 5:** Planned post hoc comparisons for all condition combinations and each hemisphere. P-values are corrected for multiple comparisons by false discovery rate. Depending on the significance of the test, either Bayes factors in favor of the alternative (*p*-value <= 0.05, given in bold) or the null hypothesis (p-value > 0.05) are reported

Comparison	Mean (μV/mm^2^)	STD (μV/mm^2^)	Range (μV/mm^2^)	df	*t*-value	*p*-value	*Cohens-d*	*BF*_*01*_*/BF*_*10*_
Contralateral								
TLDV – TL	0.07	1.14	-4.75–2.46	48	0.43	0.67	0.01	5.9
TLDV – DLTV	-0.19	1.42	-3.31–3.9	48	-0.95	0.4	0.03	4.2
TLDV – DL	-0.52	1.9	-6.22–2.88	48	-1.9	0.1	0.08	1.2
TL – DLTV	-0.26	1.61	-2.76–5.13	48	1.1	0.32	0.04	3.5
TL – DL	-0.59	2.18	-6.08–5.69	48	-1.9	0.1	0.09	1.2
DLTV – DL	-0.33	1.81	-5.69–3.77	48	-1.3	0.28	0.05	3
Ipsilateral								
TLDV – TL	-0.26	1.12	-4.36–1.51	48	-1.6	0.17	0.04	2
TLDV – DLTV	1.81	1.92	-1.29–6.64	48	6.6	**<0.001**	0.27	435200
TLDV – DL	0.86	2.47	-7.56–6.18	48	2.4	**0.038**	0.13	2.2
TL – DLTV	2.07	2.04	-0.38–8.01	48	7.1	**<0.001**	0.3	2313100
TL – DL	1.11	2.29	-4.76–7.13	48	3.4	**0.004**	0.17	22.1
DLTV – DL	-0.95	2.51	-7.84–5.6	48	-2.7	**0.02**	0.14	3.6

*TLDV* target lateral, distractor vertical, *DLTV* distractor lateral, target vertical, *TL* target lateral only*, DL* distractor lateral

## Discussion

In this study, the saliency of target and distractor stimuli were manipulated in a standard four-item search display. Behavioral and electrophysiological measures of attentional allocation were obtained in two groups of participants who viewed search displays that differed in the relative saliency of targets and distractors. The first, salient distractor (SD) group searched for a non-salient green diamond target presented among other green non-target shapes (circles) and were confronted on some trials with a salient singleton distractor (an orange square). The second, salient target (ST) group searched for a salient target (orange square) and was confronted with a non-salient green diamond distractor having the same color as the non-target (filler) circles.

In general, participants in the ST group were about 40 ms faster in responding to the search targets than participants in the SD group, showing the successful manipulation of target saliency through color. Reaction times were also faster for targets at lateral compared to vertical positions, replicating the finding of our previous study (Forschack et al., 2022a, 2022b). This result is consistent with other studies that demonstrated better left/right than upper/lower visual field processing (Abrams et al., 2012; Benson et al., 2021) and was also mirrored in the percentage of correct responses in the present study. Given these marked differences, caution should be exercised in averaging across horizontal and vertical target positions, especially when the number of target positions is not balanced between horizontal and vertical presentations. Therefore, in the present study, distractor-related costs were analyzed only when the target was in a lateral position (i.e., TL vs. TLDV).

Based on that comparison, there was no sign of behavioral costs due to distractor presence in either group with regard to percent correct responses. Furthermore, reaction times were comparable when a low salient distractor was present or when it was absent in the ST group, which is consistent with previous studies suggesting that successful distractor rejection is indicated by the absence of distractor presence costs or distractor presence benefits (Gaspelin et al., 2015; Gaspelin & Luck, 2018a, 2018b). However, for the SD group there was a small but significant difference in reaction times when the distractor was present (TLDV) compared to when it was absent (TL), such that reaction times were, on average, about 6 ms slower in the TLDV condition (see Table 2). This suggests that the salient distractor has captured attention and produced a small deleterious effect on target processing. Together, these behavioral results indicate that distractor saliency can offset top-down guidance of attention (Belopolsky et al., 2010; Folk & Remington, 1998; Lamy et al., 2004; Müller et al., 2009).

As expected, the targets in both groups elicited a typical N2pc component with an amplitude maximum over contralateral occipito-parietal scalp. The latency of the N2pc was about 50 ms earlier in the ST group, consistent with the observed group difference in reaction times (~40 ms) to the targets and reflecting the greater speed and efficiency in the selection of the highly salient target singleton. Interestingly, overall N2pc amplitudes to targets were similar in the two groups despite the marked difference in target saliency. It is conceivable that sensory imbalances due to the salience manipulation of the current study affected the target-related N2pc latency. These effects are largely consistent with a recent eye-tracking study showing faster saccades with salient compared to less salient stimuli (van Heusden et al., 2021) and ERP studies reporting earlier negativities for more salient targets (Brisson et al., 2007; Töllner et al., 2011) that translated into faster reaction times. Thus, attentional target selection speed, as indicated by reaction time, varies as a function of stimulus saliency, which is reflected in differential encoding rates in the pre-attentive bottom-up drive of visual perception (Krummenacher et al., 2001; Töllner et al., 2011). An alternative explanation that differences in sensory input (different lateralized stimulus intensities of colors in the ST group vs. same lateralized colors in the SD group) giving rise to these effects is unlikely as the luminance of the area-matched shapes was equalized on an individual basis. This procedure minimized potential sensory imbalances due to differential sensory input of the lateralized target and filler stimuli between the groups. Differential sensory input, for example, due to lateralized stimulus intensity or differential chromatic–achromatic information, usually results in early contra-ipsilateral P1 amplitude differences (Di Russo et al., 2002; Forschack et al., 2022a; Hickey et al., 2009; Mangun & Hillyard, 1991), which were absent in the current target ERP data. Interestingly, only the target but not the distractor ERP was affected by the saliency manipulation (see below), suggesting that target and distractor-related processes are functionally independent.

For both groups, the lateral singleton distractor elicited a typical Pd that was significantly different from zero at around 200 to 220 ms after stimulus onset (Fig. 2c, d; Fig. 3c, d; Fig. 4c, d), and, interestingly, without a marked latency shift between the two groups as was the case for the target-evoked N2pc as mentioned above. Furthermore, Pd amplitude comparisons between groups failed to find any significant difference. However, there seemed to be a late N2pc following the Pd evoked by the salient distractor that was absent for the non-salient distractor. The elicitation of N2pc indicates that the salient distractor captured attention, which could account for the slower reaction times when the salient distractor was present. Thus, the current results stress the role of distractor saliency and its potential to interfere with top-down guidance of attention towards the target (Feldmann-Wüstefeld, 2021; Lamy, 2021; Lamy et al., 2004). Yet, distractor saliency does not modulate the Pd, potentially hinting at separate and independent mechanisms for target and distractor processing (Chang & Egeth, 2019; Feldmann-Wüstefeld, 2021; Liesefeld et al., 2021b; Luck et al., 2021; Noonan et al., 2016; Stilwell et al., 2022). In contrast, other studies associated the Pd with behavioral costs (Burra & Kerzel, 2013; Gaspar & McDonald, 2014) and observed that it emerged after the N2pc, suggesting that attention was first deployed to the distractor and then suppressed subsequently (Feldmann-Wüstefeld et al., 2016; Kiss et al., 2012; Sawaki et al., 2012). Similarly, the present observation of an early contralateral negativity (N1pc, associated with attentional deployment; see Introduction) preceding the Pd elicited by the non-salient distractor is at odds with the notion that the Pd is a sign of *proactive* suppression (Gaspelin & Luck, 2018b; Luck et al., 2021; Stilwell et al., 2022) that prevents the distractor from capturing attention as an all-or-none phenomenon (Feldmann-Wüstefeld et al., 2021; Forschack et al., 2022a; Kerzel et al., 2021; Liesefeld et al., 2021a). Furthermore, the Pd amplitude was even reported to decrease when proactive distractor suppression was facilitated with the availability of foreknowledge about the upcoming distractor location (van Moorselaar et al., 2020; van Moorselaar & Slagter, 2019) and correlated negatively with high pre-stimulus alpha-band amplitudes in fast response trials (van Zoest et al., 2021).

What then is the functional significance of the Pd? According to previous research (Hilimire et al., 2012; Kiss et al., 2012), the emergence of the Pd seemed to critically depend on the concurrent presentation of a target along with the distractor. This is consistent with the idea that the Pd resolves stimulus competition between a target and a distractor by suppressing distractor features. Following this line of reasoning, the presentation of a distractor alone, i.e., without the task-relevant target, should not evoke a Pd. Here, however, we observed a Pd elicited by a lone distractor (DL condition) that was, interestingly, even greater in amplitude than the Pd elicited under target-distractor competition (DLTV condition). These differing results may result from differences in experimental design between the previous studies and the current experiments. In the DL condition in Hilimire et al. (2012), the distractor was presented without any additional stimuli, which reduced the complexity of the display. Therefore, attentional demands and thus, the need to reject the distractor might not be comparable with the present study where the distractor was always accompanied by “fillers.” The color singleton distractor in Kiss et al. (2012) had the same shape as the other (filler) stimuli in the display, while participants were searching for a shape singleton target. In contrast, the singleton distractors of the current experiments had a different shape than the other three additional task-irrelevant fillers in both experimental groups, while participants were also searching for a shape singleton target. This increased target-distractor similarity potentially required more attentional resources to disambiguate the distractor singleton from the filler stimuli while searching for the target when it was actually absent. Thus, it seems that Pd may indicate a general feature disambiguation process in the search for target features stored in working memory and was absent in the study of Kiss et al. (2012) due to the increased similarity between distractor and filler stimuli. Interestingly, and as discussed above, distractor saliency, i.e., local feature contrast of the distractor relative to the surrounding stimuli (Nothdurft, 1993), did not affect Pd’s amplitude, which is consistent with the results by Stilwell et al. (2022). All it may potentially require for the Pd to emerge is a perceptually competitive stimulus setting (see also (Feldmann-Wüstefeld et al., 2021) for effects of spatial distance) where the singleton distractor stimulus is defined by search-relevant feature dimensions (here shape and color) that are required to differentiate the target from the non-target stimuli. However, further studies are needed to test this prediction directly.

Further insight into the allocations of attention to the different stimuli in this search task was obtained by recording modulations of the ongoing alpha-band activity following the presentation of the search displays. Previous research has shown for different modalities that directing attention to a lateralized stimulus is associated with a relative decrease in the occipital alpha amplitude (event-related desynchronization: ERD) over the contralateral hemisphere, while suppression of a lateralized stimulus is accompanied by a relative increase of the contralateral alpha amplitude (Antonov et al., 2020; Bacigalupo & Luck, 2019; Frey et al., 2014; Gundlach et al., 2020; Klimesch, 2012; Payne et al., 2013; Sauseng et al., 2005; Thut et al., 2006; van Diepen et al., 2016; van Moorselaar & Slagter, 2019; Wöstmann et al., 2019; Zhigalov & Jensen, 2020). Here a sharp phasic decrease in alpha amplitude (ERD) was observed over both hemispheres following all displays in both the ST and SD groups. This general alpha ERD appears to indicate that attention was directed to the entire stimulus array (Bacigalupo & Luck, 2019) and is in line with the proposal of alpha amplitude (inversely) reflecting cortical excitability (Klimesch, 2012; Romei et al., 2008; Samaha et al., 2017).

When a target was presented in a lateral position in the present study, the occipital alpha ERD was substantially greater over the hemisphere contralateral to the target’s position. This pattern of lateralized desynchronization has been widely interpreted as signifying an allocation of attention to a stimulus in the contralateral visual field (Bacigalupo & Luck, 2019; Forschack et al., 2022a; Hanslmayr et al., 2011; Klimesch, 2012; Schneider et al., 2022; Van Diepen et al., 2019). Interestingly, this strongly lateralized ERD associated with target processing did not differ between the ST and SD groups, indicating a similar allocation of attention to targets of high and low saliency.

When a distractor was presented in a lateral position, however, the alpha amplitude over the contralateral hemisphere did not show an increase in amplitude with respect to the pre-stimulus baseline, nor was it enlarged with respect to the amplitude over the ipsilateral hemisphere nor relative to task-irrelevant filler stimuli (see OSM*)* in either group. In light of previous studies showing lateralized alpha increases to be a neural signature of contralateral stimulus suppression (Frey et al., 2014; Payne et al., 2013; Rihs et al., 2007; van Diepen et al., 2016; Worden et al., 2000; Wöstmann et al., 2019), this pattern of alpha modulation suggests that neither the salient nor the non-salient distractor stimuli were being actively suppressed in the present study (Foxe & Snyder, 2011; Jensen & Mazaheri, 2010). Indeed, when lateral distractors were presented alone, there was actually a greater reduction in alpha amplitude (ERD) over contralateral versus ipsilateral hemispheres, suggesting an allocation of attention to the distractor. When a lateral distractor was presented with a vertical target, however, there was no difference in the ERD over the contralateral and ipsilateral hemispheres, suggesting that attending to the target may have prevented an allocation of attention to the distractor. Again, there was no significant difference between the two groups in the alpha ERD following distractors (see Table 4), indicating that alpha lateralization was not modulated by stimulus saliency.

Based on the above-cited previous studies, we expected alpha modulations following distractors to show contralateral enhancement associated with stimulus suppression either concurrently with or as a consequence of the emergence of the Pd. If Pd reflects *proactive* distractor suppression preventing attentional capture (Gaspelin & Luck, 2018b; Luck et al., 2021; Stilwell et al., 2022), the attentional focus should be biased to locations other than the distractor position, which, in turn, would be reflected in lateralized alpha-band activity. Here we showed significantly greater alpha lateralization subsequent to target as compared to distractor presentations with comparable contralateral ERD across stimulus conditions but increased ipsilateral alpha in the target lateral conditions that potentially reflects the processing of the contralateral filler. This suggests that attention was focused on the target at the expense of the filler stimuli, whereas attentional weights were equally distributed between the distractor and the corresponding filler. Thus, reduced ipsilateral alpha to distractors could point to a relatively increased attentional deployment to context elements, i.e., the contralateral fillers (see Kerzel & Burra, 2020). However, when comparing displays containing a lateral distractor without target to the display that only contained filler stimuli (four green circles, OSM Figs. 3D and 4D), there was no sign of an ipsilateral alpha ERD, which weighs against the proposal of a serial scanning process to identify stimuli at lateral positions but suggests similar attentional deployment towards the distractor and the filler stimuli (see Forschack et al., 2022a, 2022b, for a similar argumentation).

For the evaluation of neural signals associated with either enhancement or suppression, the choice of the baseline measure is essential (Schneider et al., 2022). In our former study (Forschack et al., 2022a, 2022b), alpha-band amplitudes contralateral to the distractor were evaluated against trials containing neutral filler stimuli that were never task-relevant and had a different color than the target, which might have triggered a pronounced disengagement of attention in these trials. Therefore, using these “filler” trials as a baseline for the evaluation of distractor suppression might have been overly conservative. In the current study, target-colored filler stimuli were either presented in the same task-relevant trial containing the distractor and the target or in separate trials that only contain fillers. Nevertheless, alpha-band amplitudes contralateral to the distractor, in any case, did not exceed alpha-band amplitudes contralateral to the target-colored filler stimuli, which is hard to explain with a mechanism of *proactive* suppression of distractor processing below the level of the filler stimuli (Gaspelin & Luck, 2018b; Tam et al., 2022). Thus, the current data adds to the amounting evidence that alpha-band amplitudes might reflect target-oriented enhancement rather than distractor suppression (Bacigalupo & Luck, 2019; Foster & Awh, 2019; Noonan et al., 2016, 2018; van Moorselaar & Slagter, 2020), and, therefore, may not provide a distinctive measure to test feature-specific distractor suppression.

It should be noted that the search displays of the current study most often consisted of two objects (target and distractor), which differed from two homogenous non-target objects (circle filler) and might have induced a singleton detection strategy (Bacon & Egeth, 1994), according to which participants would focus attention on the two shape pop-out objects rather than searching for the target feature combination of a predefined color and shape. Such a singleton detection strategy may have weakened an attentional suppression effect that was expected to induce an increased contralateral alpha-band amplitude. However, the null-effect of alpha lateralization in the DLTV condition as compared to the target lateral conditions is incompatible with this possibility as it indicates that the attentional focus was not constrained to the distractor item (see also Forschack et al., 2022a, 2022b, for similar effects in a two-item search design). Future studies might further discourage a singleton detection strategy by using search displays containing four heterogeneous shapes as in Gaspelin and Luck (2018b, Experiment 1).

Before concluding, the current study has limitations that need consideration. First, for the influence of saliency on neural distractor processing, the current study mainly reports null effects, suggesting that the offset of top-down goals by the salient distractor, which resulted in behavioral net interference, is not reflected in the EEG data. However, the between-subject variance in this group design is probably too high to uncover potential small effects of distractor saliency, as for example, on alpha-band lateralization. As reported in the OSM, a high but not low salient distractor showed a reduced contralateral alpha-band amplitude below the filler baseline, when the distractor was presented alone. However, comparing contralateral alpha-band amplitudes between groups in that condition did not result in a significant effect. Thus, within-subject designs might be more sensitive to detect an effect of distractor saliency on contralateral alpha and even the Pd (but see (Stilwell et al., 2022)). Nevertheless, the current data do indicate that the influence of stimulus saliency on the target-related N2pc is much greater than its effect on the distractor-related Pd.

A second limitation has to do with the interpretation of the Pd component. The current results provide evidence against the interpretation of the Pd component as an index of *proactive* distractor suppression, i.e., the prevention of attentional capture (Gaspelin & Luck, 2018b; Luck et al., 2021; Stilwell et al., 2022), but the question of whether it reflects some form of suppression of the distractor is more difficult to answer. This question largely depends on how the term suppression is defined. If suppression is defined in terms of an absence of net distractor interference measured by behavioral performance, the presence of the Pd in the SD group that showed attentional capture by the distractor is clearly at odds with the idea of the Pd reflecting suppression in an all-or-nothing sense. Also, if the term suppression is defined as decreased distractor-related neural activity relative to a proper baseline condition, the current alpha-band results militate against the interpretation of suppression as reflected by the Pd and rather speak for disengagement of attention from the distractor in service of detecting the target (Forschack et al., 2022a; van Moorselaar & Slagter, 2020). However, one could also argue that an absence of above filler enhancement as indicated by the alpha results is well in line with the Pd reflecting distractor-related suppression as the alpha-band modulation occurs relatively late and might reflect activity at a later processing stage showing the consequence of an early stage distractor suppression (Antonov et al., 2020; Gundlach et al., 2020; Zhigalov & Jensen, 2020).

To sum up, in a typical additional singleton paradigm, we (a) manipulated the saliency of the target/distractor stimuli relative to adjacent task-irrelevant display items by varying their color and shape and (b) encouraged top-down control by never changing target and distractor identity throughout the experiment for the individual participant. Target saliency affected the speed of target processing as reflected in reaction times and the latency of the N2pc component. On the other hand, the results show that, under these conditions, top-down guidance of attention is still influenced by stimulus saliency resulting in behavioral costs when a highly salient distractor is present. Neurally, the distractor-evoked Pd was triggered independently of distractor saliency and emerged together with signs of attentional capture (i.e., together with the N1pc or N2pc). Thus, top-down proactive feature suppression in a narrow sense, i.e., preventing capture by suppressing feature values that are divergent from the target, is unlikely to be indexed by the Pd. The Pd might rather reflect a general feature disambiguation process in service of identifying the target. This process could be suppressive in some aspect and might deploy attentional resources as a function of search-relevant feature dimensions that are present during visual search. Furthermore, the observed alpha-band effects were not suggestive of attentional suppression of distractors. However, we would stress the need for clear definitions regarding the nature of suppressive mechanisms when analyzing either behavioral or neural correlates of such mechanisms. Cross-validating different measures related to visual perception like ERPs, alpha-band activity, or steady-state visual evoked potentials (Forschack et al., 2022a, 2022b) might inform a more complete picture about cognitive top-down control in the presence of salient distractors.

## Supplementary information

ESM 1 (DOCX 4.08 mb)
