# Supplementary material for: Attentional capture is modulated by stimulus saliency in visual search as evidenced by event-related potentials and alpha oscillations
Source: Atten Percept Psychophys. 2022 Dec 16;85(3):685–704. doi: 10.3758/s13414-022-02629-6 (PMC10066093; doi:10.3758/s13414-022-02629-6)
Supplement: Supplementary file 1 — (DOCX 4.08 mb) [file 13414_2022_2629_MOESM1_ESM.docx]

Supplementary Material for

Attentional capture is modulated by stimulus saliency in visual search as evidenced by event-related potentials and alpha oscillations

**Norman Forschack(1)*, Christopher Gundlach(1), Steven Hillyard(2), and Matthias M. Müller(1)**

1. Experimental Psychology and Methods, Wilhelm Wundt Institute for Psychology, University of Leipzig, Germany
2. University of California, San Diego, and Leibniz Institute of Neurobiology, Magdeburg

*Corresponding author

# Singleton interference as measured by reaction time

To evaluate potential perceptual costs of the singleton distractor for each group, singleton present versus absent conditions were compared. As written in the main text, differential singleton distractor interference was observed for reaction time data. To illustrate this effect, *Supplementary Figure 1* depicts the reaction times for each participant and salience group and the respective means when the singleton distractor was present or absent.


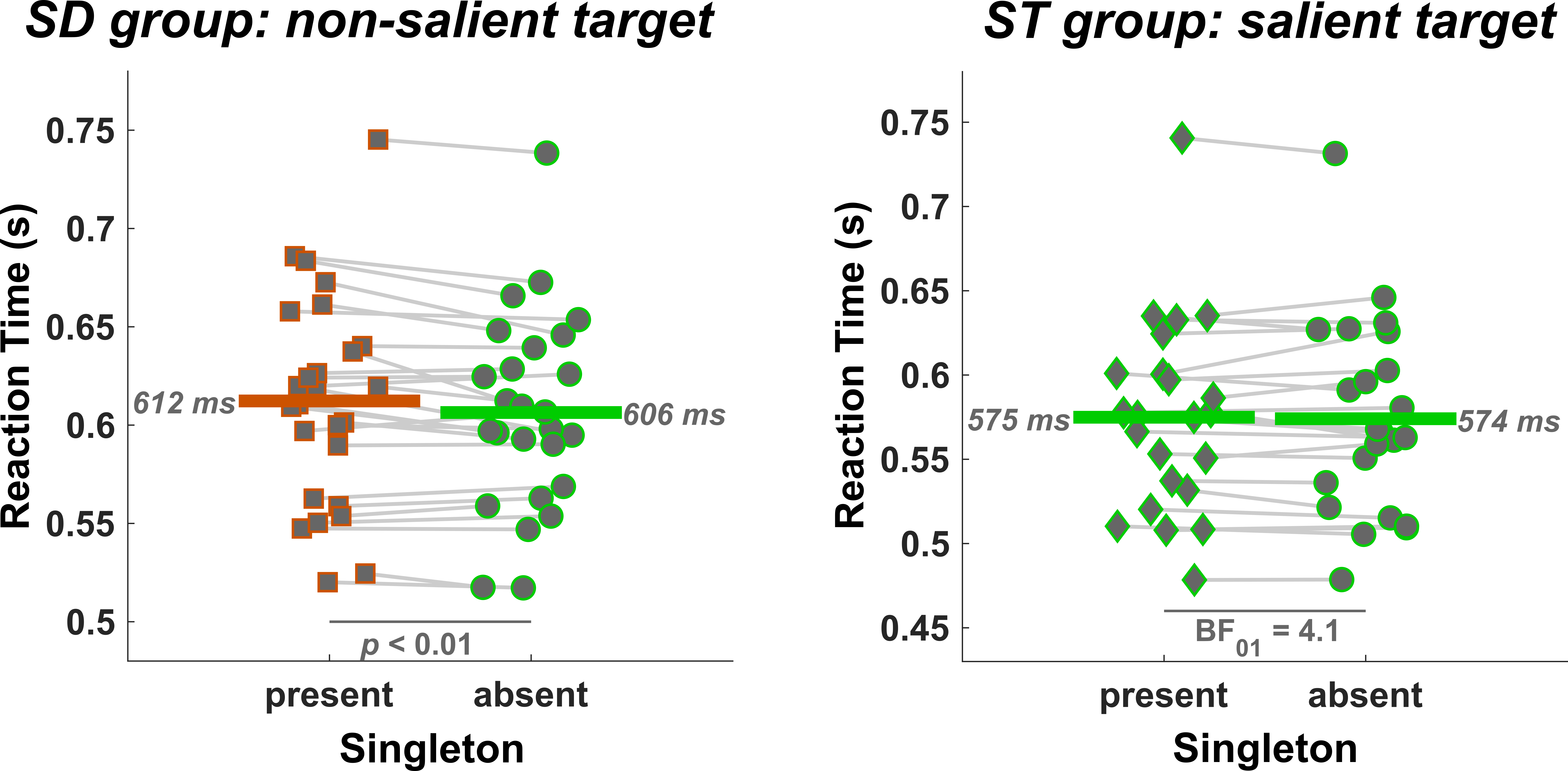


Supplementary Figure 1. Comparison of individual reaction times averaged for singleton present (TLDV) and singleton absent (TL) conditions, separately for the salient distractor (SD) and salient target (ST) group. The color and form of the elements indicate the color and form of the singleton and non-singleton distractors, i.e., an orange square or green circle in the SD group and a green diamond or circle in the ST group. Colored bars depict the sample average for each condition, respectively.

There was a small but consistent speedup of reaction times when the salient singleton was absent in the SD group but there was no difference in reaction times in the ST group, irrespective of whether the less salient singleton was present or not.

# Selection of ERP time windows

As described in the main text, ERPs contra- and ipsilateral to target and distractor stimuli were averaged across participants, and ERP windows were centered at the grand-average N1 and the two subsequent peaks, here labelled P2 and N2. *Supplementary Figure 2* shows this grand-average ERP were target and distractor lateral trials have been pooled. The average potentials extracted from the ERP time windows were used to quantify the amplitude of the difference potentials related to the cardinal peaks of the N1-P2-N2 complex, i.e., the N1pc, the Pd, and the N2pc, respectively.





Supplementary Figure 2. Grand-average ERP contra- and ipsilateral to pooled target and distractor lateral trials. Grey boxes cover component peaks of the N1-P2-N2 complex and indicate average ERP time windows used to quantify the N1pc, Pd and N2pc difference potentials.

# Filler condition-corrected alpha-band amplitude time courses

The alpha-band laterality effect reported in the main text, i.e., a greater ERD asymmetry for target than distractor stimuli, independent of stimulus saliency, seems to originate from variance of the ipsilateral signal, i.e., alpha-band modulation contralateral to the filler stimuli that appeared contralateral to the target/ distractor (see *table 5* of the main text). However, such a conclusion would be premature, as it disregards a potential modulation originating from a signal reflecting the bilateral stimulus onset. To account for this, conditional alpha-band amplitudes were referenced to alpha-band activity triggered by the filler-only display (i.e., four green circles). Similar to *Figures 5* and *6* of the main text, S*upplementary Figures 3* and *4* show the resulting alpha-band amplitude time courses for each condition for the SD and ST groups, respectively, after subtracting the time courses elicited by the filler-only display.


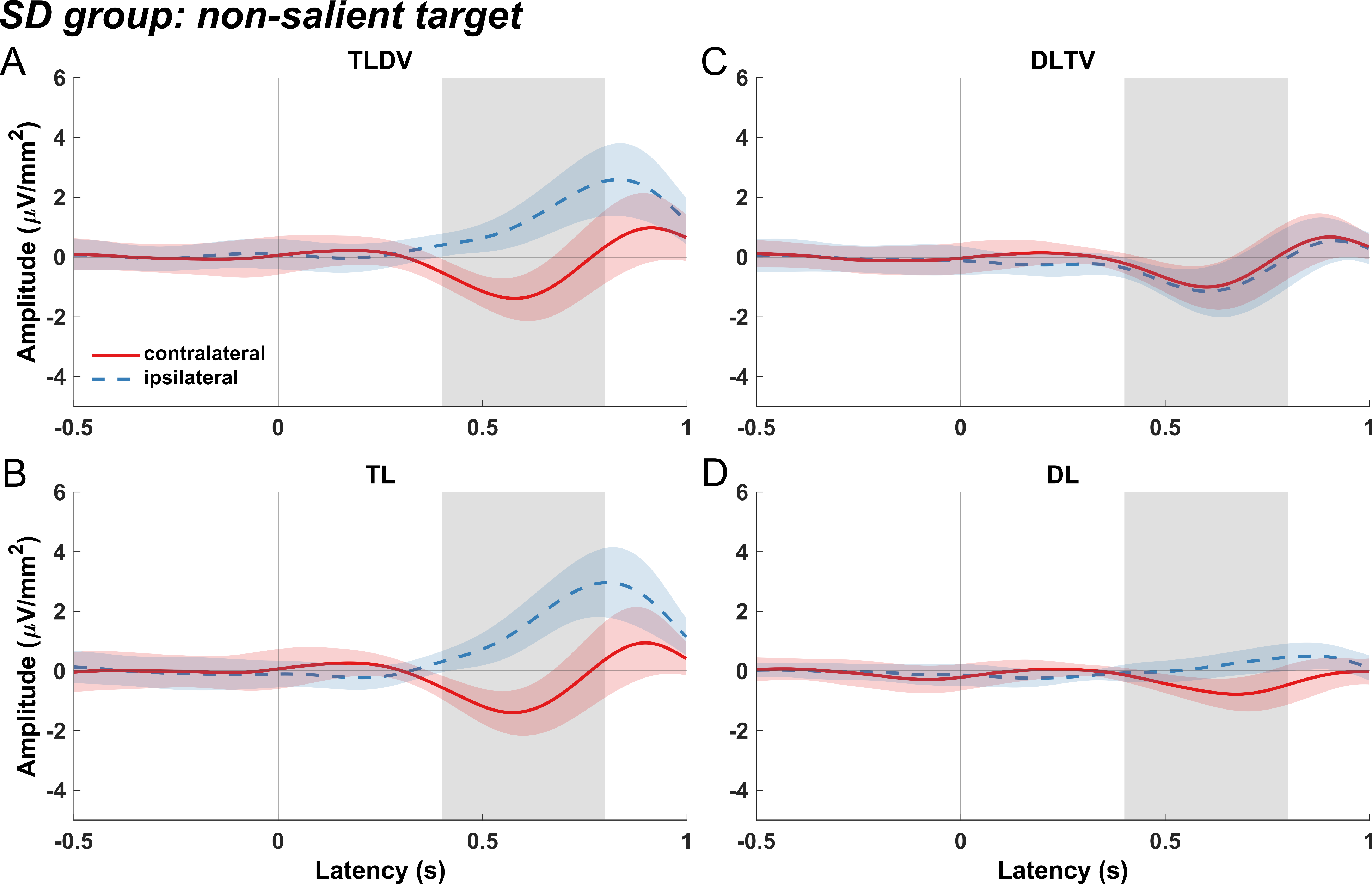


Supplementary Figure 3. Contra- and ipsilateral alpha source current density time courses for the SD group with a non-salient target and a salient singleton distractor relative to alpha-band activity of filler-only displays. Mean values were extracted at the symmetrical electrode clusters used for the main analysis for the conditions "target lateral - distractor vertical" (A, TLDV), "single target lateral" (B, TL), "distractor lateral - target vertical" (C, DLTV) and "single distractor lateral" (D, DL). Colored shaded areas show within-subject confidence intervals. The difference between contralateral and ipsilateral amplitudes was compared in the time range from 400 to 800 ms relative to stimulus onset indicated by the grey boxes. Zero marks the onset of the visual search display.





Supplementary Figure 4. Contra- and ipsilateral alpha source current density time courses for the ST group with a salient target and a non-salient singleton distractor. Other figure conventions are the same as those in Supplementary Figure 3.

Notably, the overall pattern of results remained. First, there was no increase of alpha-band amplitudes contralateral to distractor stimuli relative to ipsilateral alpha-band amplitudes. Second, statistical modeling and comparisons across conditions for the contra- and ipsilateral signal were identical as the filler condition signal was subtracted from each of the remaining conditions and thereby canceled out when calculating the condition contrasts. It should be noted, though, that the remaining ERD in the TLDV, DLTV, and TL conditions cannot be attributed to either stimulus or response-related activity conclusively, as participants were not required to respond in the filler-only condition. Furthermore, with the current design it may be difficult to disentangle whether distractor-related alpha-band modulations arose from differential lateralized distractor processing due to target presence or absence in the DLTV compared to the DL condition or whether the lateral alpha-band signals captured dynamics related to a bilateral alpha-band modulation indicating the processing of the whole stimulus set (i.e., including stimuli at the vertical positions).

# Exploratory analysis: EEG measures and reaction time

In an exploratory analysis, we investigated which neural measure best predicted the reaction time effect between the TLDV and TL conditions, i.e., which measure explained potential behavioral costs or benefits when a distractor was present. We consider this analysis to be very preliminary, because mean behavioral RT costs in the presence of the distractor were either absent in the ST group or very small (6 ms on average) in the SD group (although statistically significant). To this end, ipsilateral alpha and N2pc CSD values were contrasted between TLDV and TL for each group. Then, reaction time differences (TLDV minus TL) were modeled by linear regression with the two interacting factors, *alpha x group,* and *N2pc x Group,* plus all corresponding main effect factors. Best subset selection identified the most parsimonious model that explained the behavioral effect best. With an adjusted *R²* of 0.24, the interaction *alpha x group* (*β* = 0.0041) with the main effects *alpha* (*β* = -0.0062) and *group* (*β* = -0.0036) best explained the reaction time differences between TLDV and TL (*F*(2, 45) = 5.96, *p* = 0.002). The interaction indicates that the relationship between alpha lateralization and behavior was more pronounced in one of the two groups. In fact, regressions calculated separately for each group with alpha as a single factor revealed a significant negative relationship for the SD (*β* = -0.0062, *t*(24) = -3.4, *p* = 0.002, *adjR²* = 0.3) but not the ST group. Thus, higher ipsilateral αCSD values in the condition without the distractor (or smaller values in the TLDV condition) correlated with a greater behavioral effect, i.e., with more costs when the distractor was present. Interestingly, the identical modeling approach for contralateral alpha resulted in no significant effects whatsoever, consistent with the findings reported in Table 5 of the main text that contralateral alpha was not modulated across conditions. We find these exploratory results of interest, because they suggest that if ipsilateral alpha amplitudes were similar for the target alone (TL) compared to the competition condition (TLDV), attentional capture from the salient distractor would be reduced.
